# Complete chloroplast genome sequence of *Ardisia gigantifolia* (Myrsinaceae), a vulnerable medicinal plant

**DOI:** 10.1080/23802359.2019.1688727

**Published:** 2019-11-13

**Authors:** Yancai Shi, Bingbing Liu

**Affiliations:** a Institute of Loess Plateau, Shanxi University, Taiyuan, Shanxi, China;; b Guangxi Institute of Botany, Guangxi Zhuang Autonomous Region and Chinese Academy of Sciences, Guilin, China

**Keywords:** *Ardisia*, chloroplast genome, phylogenetic analysis

## Abstract

*Ardisia gigantifolia* (Myrsinaceae) is a perennial shrub and widely distributed in Southeast Asia. It is well known for its medicinal values and has the potential for development of novel phytopharmaceuticals. Here, we first report and characterize its complete chloroplast genome based on Illumina paired-end sequencing data. The complete plastid genome was 156,216 bp, which contained inverted repeats (IR) of 26,047 bp separated by a large single-copy (LSC) and a small single-copy (SSC) regions of 85,725 bp and 18,397 bp, respectively. The cpDNA contains 134 genes, comprising 88 protein-coding genes, 37 tRNA genes, 8 rRNA genes, and 1 processed pseudogene. The overall GC content of the plastome is 37.3%. The phylogenetic analysis of 17 selected chloroplast genomes demonstrated that *A. gigantifolia* is closely related to the congeneric *A. polysticta*.

*Ardisia gigantifolia* Stapf., a vulnerable perennial medicinal plant, is the species of the genus *Ardisia* belong to family Myrsinaceae. It is naturally distributed in Southeast Asia including southern provinces of China, Thailand, Malaysia, and Vietnam (Mu et al. 2012). It is well known for its medicinal values and has the potential for the development of novel phytopharmaceuticals. The whole plant of this species has been used in folk medicine to eliminate blood stasis, disperse swelling, improve blood circulation, and also as an analgesic (Kobayashi and de Mejia 2005). However, largely due to anthropogenic over-cutting and natural habitat’s fragmentation of species, the wild resources of *A. gigantifolia* have been dramatically decreased and need urgent protection and restoration. Herein, we first report and characterize its complete plastome based on Illumina paired-end sequencing data, which will contribute to the further studies on its genetic research and resource utilization. The annotated chloroplast (cp) genome of *A. gigantifolia* has been deposited into GenBank with the accession number MN548760.

In this study, *A. gigantifolia* was sampled from Guangxi Zhuang Autonomous Region of China, located at 107°57′01″ E, 21°48′59″ N. A voucher specimen (Y.-C. Shi et al. H045) was deposited in the Guangxi Key Laboratory of Plant Conservation and Restoration Ecology in Karst Terrain, Guangxi Institute of Botany, Guangxi Zhuang Autonomous Region and Chinese Academy of Sciences, Guilin, China. The experiment procedure is as reported in Zhang et al. (2019). Around 2 Gb clean data were used for the cp genome de novo assembly by the program NOVOPlasty (Dierckxsens et al. 2017) and direct-viewing in Geneious R11 (Biomatters Ltd., Auckland, New Zealand). Annotation was performed using the program Plann (Huang and Cronk 2015) and Sequin (http://www.ncbi.nlm.nih.gov/).

The cp genome of *A. gigantifolia* is a typical quadripartite structure with a length of 156,216 bp, which contained inverted repeats (IR) of 26,047 bp separated by a large single-copy (LSC) and a small single-copy (SSC) regions of 85,725 bp and 18,397 bp, respectively. The cpDNA contains 134 genes, comprising 88 protein-coding genes, 37 tRNA genes, 8 rRNA genes, and 1 processed pseudogene. Among the annotated genes, 15 of them contain one intron (*atp*F, *ndh*A, *ndh*B, *rps*16, *rpoC*1, *pet*B, *pet*D, *rpl*16, *rpl*2, *trn*A-UGC, *trn*I-GAU, *trn*G-UCC, *trn*K-UUU, *trn*L-UAA, and *trn*V-UAC), and three genes (*clp*P, *rps*12, and *ycf*3) contain two introns. The overall GC content of the plastome is 37.3%.

To identify the phylogenetic position of *A. gigantifolia*, phylogenetic analysis was conducted. A neighbour-joining (NJ) tree with 1000 bootstrap replicates was inferred using MEGA version 7 (Kumar et al. 2016) from alignments created by the MAFFT (Katoh and Standley 2013) using plastid genomes of 17 species. It showed the position of *A. sinensis* was close to the congeneric *A. polysticta* (Figure 1). Our findings can be further used for plastome evolution, population genomics, and phylogenomic studies of Myrsinaceae. It will also provide fundamental data for the conservation, utilization, and management of this vulnerable medicinal plant.

**Figure 1.**
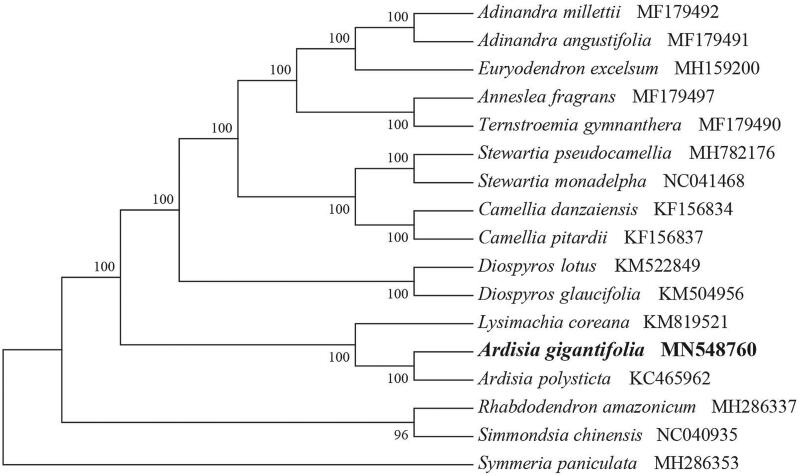
NJ phylogenetic tree of *A. gigantifolia* with 16 species was constructed by chloroplast plastome sequences. Numbers on the nodes are bootstrap values from 1000 replicates. *Symmeria paniculata* was selected as outgroups.
